# Extraordinarily large kinetic isotope effect on alkene hydrogenation over Rh-based intermetallic compounds

**DOI:** 10.1080/14686996.2019.1642139

**Published:** 2019-07-11

**Authors:** Shinya Furukawa, Pingping Yi, Yuji Kunisada, Ken-Ichi Shimizu

**Affiliations:** a Institute for Catalysis, Hokkaido University, Sapporo, Japan; b Elementary Strategy Initiative for Catalysis and Battery, Kyoto University, Kyoto, Japan; c Center for Advanced Research of Energy and Materials, Faculty of Engineering, Hokkaido University, Sapporo, Japan

**Keywords:** Kinetic isotope effect, intermetallic compound, hydrogenation, alkene, 10 Engineering and Structural materials, 106 Metallic materials, 205 Catalyst / Photocatalyst / Photosynthesis

## Abstract

A series of Rh-based intermetallic compounds supported on silica was prepared and tested in alkene hydrogenation at room temperature. H_2_ and D_2_ were used as the hydrogen sources and the kinetic isotope effect (KIE) in hydrogenation was studied. In styrene hydrogenation, the KIE values differed strongly depending on the intermetallic phase, and some intermetallic compounds with Sb and Pb exhibited remarkably high KIE values (>28). An extraordinarily high KIE value of 91, which has never been reported in catalytic reactions at room temperature, was observed particularly for RhPb_2_/SiO_2_. RhPb_2_/SiO_2_ also showed high KIE values in the hydrogenation of other unsaturated hydrocarbons such as phenylacetylene and cyclohexene. The density functional theory calculation focused on the surface diffusion of hydrogen suggested no contribution of the quantum tunneling effect to the high KIE values observed. A kinetic study revealed that the dissociative adsorption of H_2_ (D_2_) was the rate-determining step in the styrene hydrogenation over RhPb_2_/SiO_2_. We propose that the large KIE originates from the quantum tunneling occurring at the hydrogen adsorption process with the aid of the specific surface structure of the intermetallic compound and adsorbate alkene.

## Graphical abstract

**Figure:**
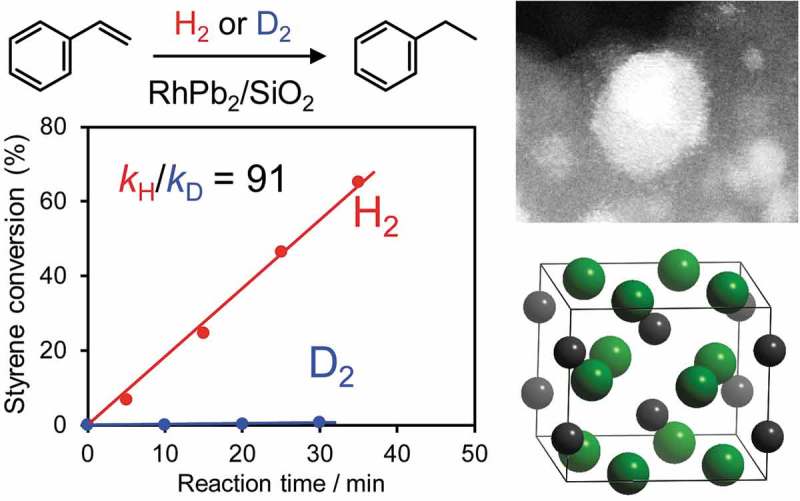

## 1. Introduction

Intermetallic compounds are stoichiometric compounds consisting of two or more metal elements with considerably different electronic characters or atomic radii (apart from each other in the periodic table) [1]. Unlike conventional solid-solution alloys, intermetallic compounds have a specific crystal structure different from those of the parent metals, thereby displaying a specific and highly ordered atomic arrangement at the surface. Intermetallic compounds have recently attracted increasing attention as promising inorganic materials for heterogeneous catalysts because of their unique surface characteristics [2–6]. For instance, such a specific surface reaction environment allows the control of adsorption configuration, diffusion behavior, and reaction dynamics of the reactant molecules [7–9]. Therefore, highly challenging molecular transformations, such as stereo- [7], chemo- [8], and regioselective [9] hydrogenation, could be done with the aid of surface stereochemistry. During the course of our attempt to discover further unique catalytic behaviors of intermetallic compounds, we observed that some Rh-based intermetallic compounds show an extraordinarily large kinetic isotope effect (KIE) on alkene hydrogenation (*k*_H_/*k*_D_ = 91). This value is remarkably higher than those typically observed in organic reactions with deuteration [10]. Although rather high KIE values (>100) have been observed at cryogenic temperatures because of the quantum tunneling effect, to the best of our knowledge, no catalytic reaction showing such a high KIE value at room temperature has been reported. We report herein on a highly specific hydrogenation behavior of some Rh-based intermetallic compounds, which runs counter to common sense in isotope chemistry.

## 2. Methods

### 2.1. Catalyst preparation

The Rh-based catalysts (Rh: 3 wt%) were prepared by pore-filling co-impregnation using silica as a support. A mixed aqueous solution of Rh(NO_3_)_3_ (Furuya Metal Co. Ltd., 4.87 wt% Rh) and the second metal salts (Bi(NO_3_)_3_∙nH_2_O (Wako, 99.9%), Ga(NO_3_)_3_∙8H_2_O (Wako, 99.9%), (NH_4_)_2_GeF_6_ (Sigma Aldrich, 99.99%), In(NO_3_)_3_∙8H_2_O (Kanto, 99%), SnCl_2_ (Kanto, 97%), and Zn(NO_3_)_2_∙6H_2_O (Kanto, 99%)) was added to dried silica gel (CARiACT G-6, Fuji Silysia, *S*_BET_ = 470 m^2^ g^−1^) such that the solutions filled the silica pores. The mixtures were sealed overnight at room temperature and dried over a hot plate. Reduction was then performed under flowing H_2_ at 800°C (600°C only for RhZn to avoid Zn sublimation) for 1 h. The atomic ratio of Rh and the second metal (Rh/M) was set to 1 (for RhM) or 0.5 (for RhM_2_).

### 2.2. Characterization

The crystal structure of the prepared catalyst was examined using powder X-ray diffraction (XRD) by a Rigaku MiniFlex II/AP diffractometer with Cu Kα radiation. The crystallite size was estimated using Scherrer equation with the Scherrer constant of 0.85 for volume weighted mean diameters. High-angle annular dark-ﬁeld scanning transmission electron microscopy (HAADF-STEM) was employed using a JEOL JEM-ARM200 M microscope equipped with an energy-dispersive X-ray (EDX) analyzer (EX24221M1G5T). STEM analysis was performed at an accelerating voltage of 200 kV. For the TEM specimen preparation, all samples were sonicated in ethanol and dispersed on a Mo grid supported by an ultrathin carbon ﬁlm.

### 2.3. Catalytic reactions

A catalyst (100 mg) was placed in a 50 mL three-necked round-bottom quartz flask equipped with a silicone rubber septum and a gas storage balloon, which was pretreated under an H_2_ stream (20 mL·min^−1^) at 400°C for 0.5 h using a mantle heater. After the pretreatment, dry N_2_ (20 mL·min^−1^) was passed into the flask to replace the residual H_2_. The flask was then cooled to room temperature. Subsequently, H_2_ or D_2_ was filled into the reactor, followed by the addition of a reaction mixture containing a solvent (THF, Kanto; 99.8%, 5 mL), alkene/alkyne (0.5 mmol), and biphenyl (TCI; 99%, 0.75 mmol) as an internal standard into the flask through the septum at 25°C. The products were identified and quantified using gas chromatography-mass spectrometry (SHIMADZU GCMS-QP2010 with an Ultra ALLOY Capillary Column UA^+^-1; Frontier Laboratories Ltd.) and a GC (Shimadzu GC-14B with an Ultra ALLOY Capillary Column UA^+^-1; Frontier Laboratories Ltd.), respectively. For alkene hydrogenation, no by-product was detected and the carbon balance ranged within 100 ± 5% in each reaction. For the kinetic study, the partial pressure of H_2_ (*P*_H2_) was changed by diluting pure H_2_ with N_2_ as an inert gas (0.5 ~ 1.0 atm, standard condition: 1.0 atm). Styrene concentration was also changed using a lower amount of the reactant (0.1 ~ 0.2 M, standard condition, 0.1 M). The reaction was performed at a constant stirring rate of 360 rpm. We confirmed that reaction rate did not change when the stirring rate was changed to 240 rpm. Therefore, the reaction is not limited by mass-transportation.

### 2.4. Computational details

Periodic density functional theory (DFT) calculations were performed using the Vienna Ab initio Simulation Package (VASP 5.4.1) [11–16] and the Perdew−Burke−Ernzerhof exchange-correlation functional [17] based on the generalized gradient approximation. The plane-wave basis set was truncated at a kinetic energy of 500 eV. A first-order Methfessel−Paxton method of 0.2 eV was adopted for smearing [18]. The reciprocal space was sampled using a *k*-point mesh of 6 × 6 × 1, as generated by the Monkhorst−Pack scheme [19]. These calculation settings were set to an energy tolerance of 3.0 × 10^−3^ eV per atom. The surfaces were modelled using metallic slabs with a (1 × 1) unit cell and a thickness of four atomic layers with 15 Å of vacuum spacing. The Rh(111), RhIn(110), and RhPb_2_(100) planes were chosen as the most stable surfaces according to the literature [8]. We determined the optimized surface structures by performing preliminary calculations in which the top two layers were relaxed until the forces on each atom were smaller than 0.02 eV Å^−1^. The most stable hydrogen adsorption configurations were determined using the supercell with one hydrogen atom and the corresponding slabs. We considered four, five, and five high-symmetric adsorption sites for the Rh(111), RhIn(110), and RhPb_2_(100) surfaces as the initial adsorption sites, respectively. The hydrogen atom and the top two layers of slabs were relaxed until the force on each atom was less than 0.02 eV Å^−1^. The adsorption energy of a hydrogen atom was defined as follows: *E*_ad_ = *E*_A−H_ − (*E*_S_ + 0.5*E*_H2_), where *E*_A−H_ is the energy of the slab together with the hydrogen atom; *E*_H2_ is the total energy of the free H_2_ molecule; and *E*_S_ is the total energy of the bare slab. The diffusion barrier of hydrogen on each surface was estimated using the climbing image nudged elastic band method [20,21]. The vibrational frequency and the zero-point energy of the adsorbed hydrogen/deuterium were calculated using the harmonic oscillator approximation. The hopping rate of the hydrogen atom on the surfaces at 300 K was calculated as follows: (quantum tunnelling) *k*_QT_ = *ν* × *q*, (thermal diffusion) *k*_DT_ = *ν* × *k*_B_, where *ν, q*, and *k*_B_ are the standard vibrational frequency, quantum tunnelling probability, and Boltzmann distribution factor for the diffusion barrier considering the zero-point energy, respectively. The quantum tunnelling probability with the corresponding kinetic energy of a hydrogen atom at 300 K was calculated from the diffusion barrier considering the zero-point energy using a local reflection/transmission matrix method [22]. H_2_ adsorption and dissociation process on the Rh-based surfaces, where the dispersion force may play the important role, were calculated using the rev-vdW-DF2 functional for the consideration of the van der Waals interaction [23].

## 3. Results and discussion

**Figure 1.**
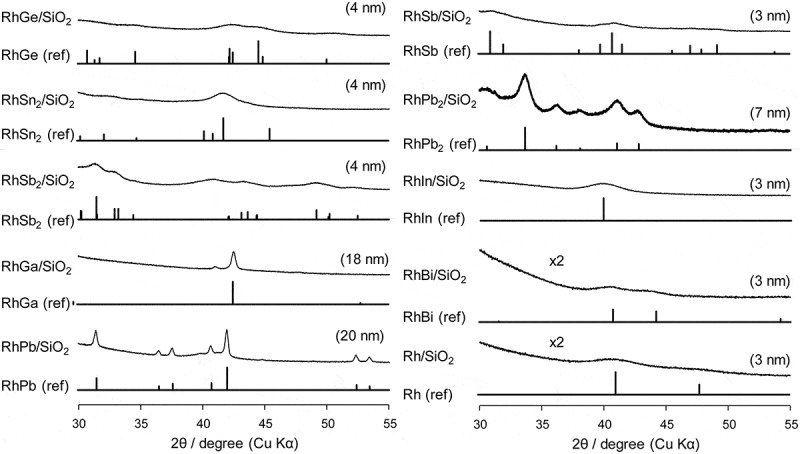
XRD patterns of supported Cu catalysts. Values in parentheses show crystallite sizes estimated by Scherrer equation.

The prepared Rh-based catalysts were analyzed by XRD such that the desired intermetallic phases were formed (Figure 1). The diffractions assigned to the desired intermetallic phase were clearly observed for each catalyst (RhGe [24], RhSn_2_ [25], RhSb_2_ [25], RhGa [26], RhPb [27], RhSb [28], RhPb_2_ [29], RhIn [30], RhBi [31], and Rh [26], see Figure S1 for wide range XRD patterns of RhGa and RhIn), suggesting that the nanoparticles of the Rh-based intermetallic compounds were formed on the SiO_2_ support. The crystallite sizes were estimated by applying Scherrer equation to the most intense diffractions. They ranged from 3 to 7 nm with a high metal dispersion in most catalysts, but were as large as 18 and 20 nm in RhGa and RhPb, respectively.

**Figure 2.**
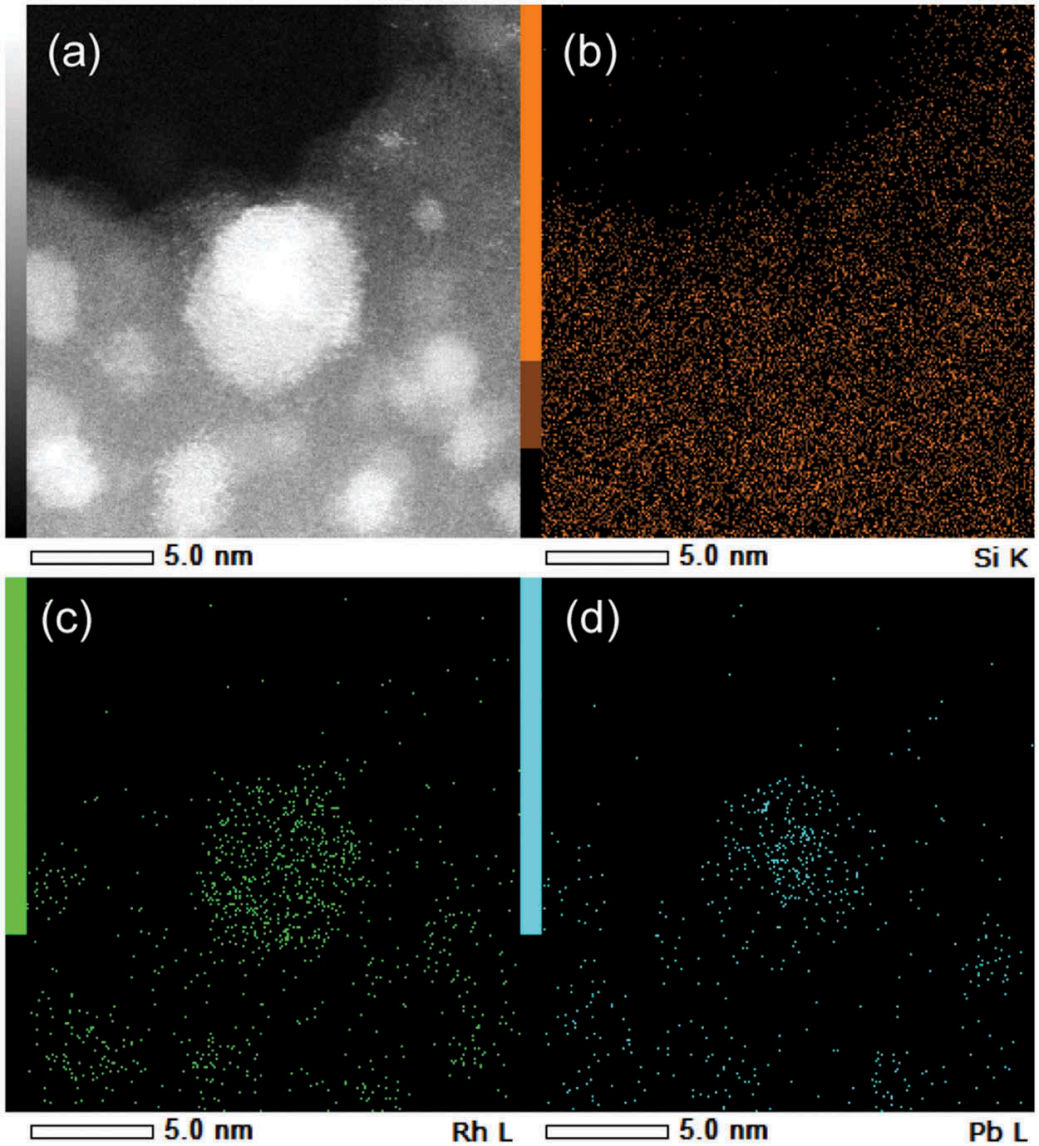
(a) HAADF-STEM image of RhPb_2_/SiO_2_ catalyst and the corresponding elemental maps of (b) Si, (c) Rh, and (d) Pb acquired using EDX.

Figure 2(a) shows the HAADF-STEM image of the RhPb_2_/SiO_2_ catalyst. Small nanoparticles with 3–6 nm sizes were observed, which were roughly consistent with the XRD result. The elemental maps of Rh and Pb acquired using the EDX analysis on this field revealed that the Rh and Pb atoms composing the nanoparticles were homogeneously dispersed (Figure 2(c,d)). This result suggests that the observed nanoparticles comprised both Rh and Pb without segregation. The EDX mapping clearly showed that the Rh–Pb nanoparticles were placed on the SiO_2_ support (Figure 2(b)).

**Figure 3.**
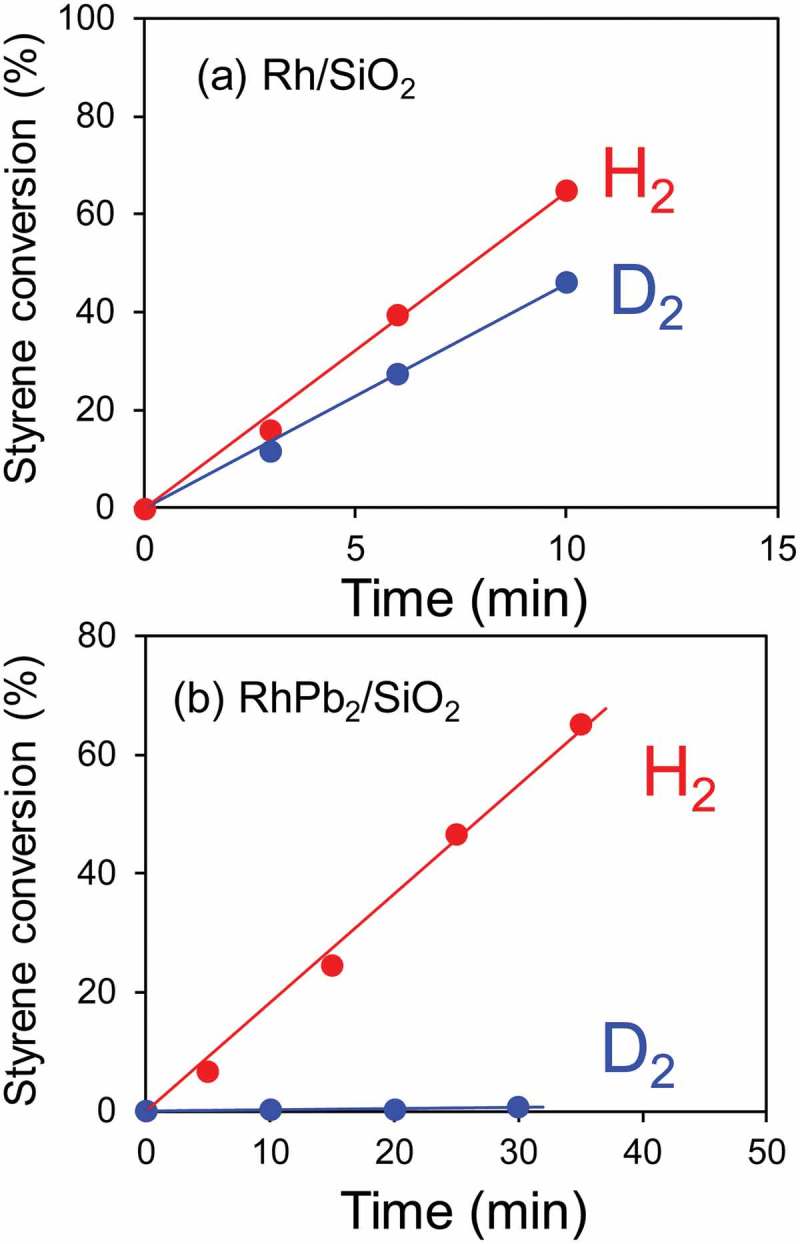
Time-course of ethylbenzene yield in styrene hydrogenation over (a) Rh/SiO_2_ and (b) RhPb_2_/SiO_2_ catalysts when H_2_ (red) or D_2_ (blue) was used as a hydrogen source.

The prepared Rh-based catalysts were then tested for styrene hydrogenation as a model reaction. The reaction was performed in the liquid phase in the presence of 1 atm H_2_ or D_2_ at room temperature. Figure 3(a) shows the time-course of the styrene conversion when a monometallic Rh/SiO_2_ was used as the hydrogenation catalyst. The styrene conversion increased linearly with time, where the reaction rate was estimated as 32.3 and 22.9 μmol min^−1^ when H_2_ and D_2_ were used as the hydrogen sources, respectively. We confirmed that the product in the hydrogenation under D_2_ was ethylbenzene-d2, demonstrating that D_2_ truly acted as the hydrogen source in this reaction. The KIE value (*k*_H_/*k*_D_) was estimated as 1.4, indicating that no apparent primary KIE was observed for the monometallic catalyst. Rh-based intermetallic catalysts were also tested in a similar manner. Table 1 summarizes the corresponding reaction rates and the KIE values. In most cases, *k*_H_/*k*_D_ was much higher than unity, showing the contribution of the large KIE. Remarkably high KIE values were observed particularly for the Pb- and Sb-containing intermetallic compounds. Only a trace amount of styrene was converted in the hydrogenation over RhPb_2_ (Figure 3(b)), resulting in an extraordinarily high KIE value of 91. We confirmed the reproducibility for RhSb and RhPb_2_, where similar high KIE values were obtained (Table 1, values in parentheses; see also Figure S2 for the time-course of styrene conversion). We also tested other unsaturated hydrocarbons, such as phenylacetylene, cyclohexene, and neopentane, in a similar manner (Table 2). The monometallic Rh/SiO_2_ showed KIE values close to unity for all the substrates tested, reflecting that no KIE appeared in the hydrocarbon hydrogenation. On the contrary, RhPb_2_/SiO_2_ also exhibited large KIE values for these substrates. The KIE value observed for the styrene hydrogenation was much higher than those observed in the conventional organic molecular transfers with deuterium at room temperature, which were typically below 20 [10]. Therefore, a specific effect might contribute to the hydrogenation system of RhPb_2_. A possible interpretation for this is the involvement of quantum tunneling, which typically showed a large KIE in the chemical reaction with deuterium. A KIE value higher than 100 was also reported in the hydrogen diffusion process over a solid surface at a cryogenic temperature [32].

**Table 1.** Summary of reaction rates and *k*_H_/*k*_D_ values.*^a^*

	*r*_H_	*r*_D_	
Catalyst	(μmol min^−1^)		*k*_H_/k_D_
RhGe	1.08	2.02	0.5
RhSn_2_	0.38	0.72	0.5
RhZn	17.2	24.0	0.7
Rh	32.3	22.9	1.4
RhBi	3.36	0.85	3.9
RhIn	1.02	0.22	4.7
RhGa	12.5	1.21	10
RhPb	5.68	0.21	27
RhSb_2_	1.51	0.04	40
RhSb *^b^*	2.55 (3.90)	0.055 (0.11)	45 (36)
RhPb_2_ *^b^*	9.15 (9.55)	0.10 (0.12)	91 (80)

*^a^ r*_H(D)_: conversion rate of styrene when H_2_(D_2_) was used as a hydrogen source. *^b^* Values in parentheses indicate results obtained in another catalytic run (reproducibility).

**Table 2.** KIE values obtained in hydrogenation of various unsaturated hydrocarbons.

Reactant	*k*_H_/*k*_D_	
	Rh/SiO_2_	RhPb_2_/SiO_2_
Styrene	1.4	91
Phenylacetylene	1.8	26
Cyclohexene	0.8	16
Neohexene	1.0	−

**Figure 4.**
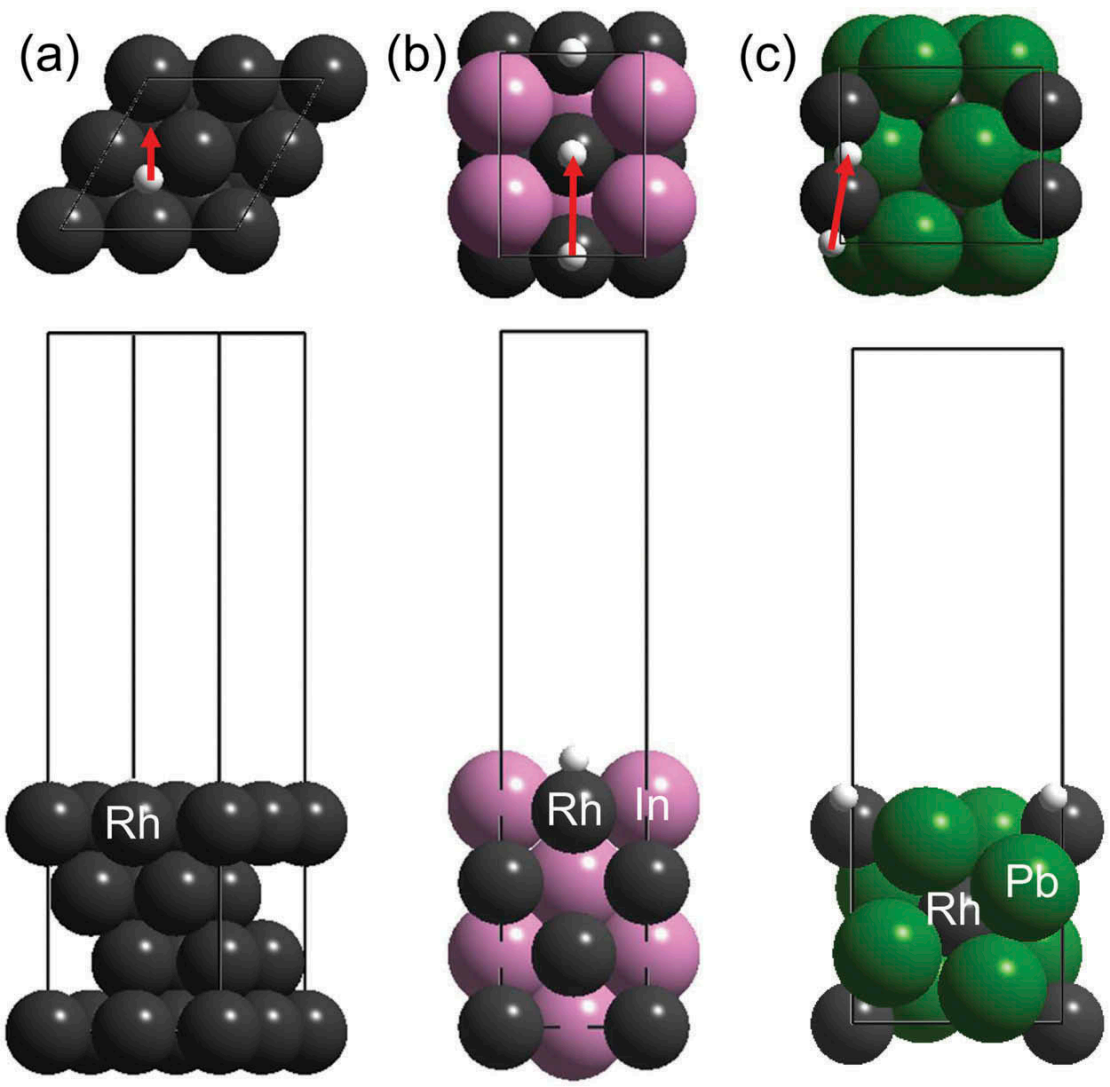
Slab structures of (a) Rh(111), (b) RhIn(110), and (c) RhPb_2_(100) and diffusion paths of a hydrogen atom (red arrows).

Therefore, we then focused on the hydrogen diffusion process on the surface of the intermetallic compounds with respect to the quantum tunneling effect. We focused on the three materials showing no (Rh: 1.4), moderate (RhIn: 4.7), and very high KIE (RhPb_2_: 91) values. The corresponding most stable surfaces (Rh(111), RhIn(110), and RhPb_2_(100)) were chosen for the DFT study. Figure 4 shows the slab structures of the target Rh-based surfaces and the diffusion paths of a hydrogen atom between stable adsorption sites. The diffusion paths along with the Rh rows were considered for bimetallic surfaces. The hydrogen adsorption on each surface was exothermic without a significant difference in the adsorption energy (*E*_ad_, Table 3). Rh(111) and RhIn(110) showed similar diffusion barriers (*E*_dif_) that were sufficiently lower than those of typical chemical reactions. RhPb_2_(001) exhibited a *E*_dif_ that was much higher than those of Rh(111) and RhIn(110). We calculated the hopping rates of a hydrogen/deuterium atom on these surfaces based on quantum tunneling (*k*_QT_) or thermal diffusion (*k*_TD_) (Table 3). The ratios of *k*_QT_ for H and D were in the following order: Rh (3.3 × 10^3^) < RhIn (2.5 × 10^5^) ≪ RhPb_2_ (1.9 × 10^17^). The large difference in *k*_QT_ for RhPb_2_ may stem from the larger height and width of the diffusion barrier, which significantly enlarged the difference of the quantum tunneling probabilities of H and D. However, the *k*_TD_ on RhPb_2_ was much higher by several orders of magnitude than *k*_QT_ for each surface. Therefore, the thermal hopping of the hydrogen atoms could likely occur easily at room temperature and contribute dominantly to the overall surface diffusion rate. In the case of the thermal diffusion, no significantly high KIE was estimated even when the zero-point energy difference of surface H and D was considered. Therefore, the large KIE observed did not originate from the surface diffusion process. We note that even if we considered the dispersion force using the rev-vdW-DF2 functional [23], the adsorption energy and diffusion barrier of the hydrogen atom on each surface changed only by approximately 10 meV. Thus, the dispersion force does not contribute to the surface diffusion process.

**Table 3.** Adsorption energy (*E*_ad_), diffusion barrier (*E*_dif_), and hopping rate of a hydrogen atoms on Rh-based surfaces considering quantum tunneling (*k*_QT_) and thermal diffusion (*k*_TD_).

		Rh	RhIn	RhPb_2_
*E*_ad_/eV		−0.54	−0.37	−0.68
*E*_dif_/eV		0.12	0.12	0.64
*k*_QT_	H/s^−1^	5.7 × 10^5^	3.8 × 10°	2.5 × 10^−27^
	D/s^−1^	1.7 × 10^2^	1.5 × 10^−5^	1.3 × 10^−44^
	H/D	3.3 × 10^3^	2.5 × 10^5^	1.9 × 10^17^
*k*_TD_	H/s^−1^	5.3 × 10^11^	4.1 × 10^11^	3.8 × 10^3^
	D/s^−1^	2.9 × 10^11^	2.0 × 10^11^	1.6 × 10^3^
	H/D	1.9	2.1	2.4

**Figure 5.**
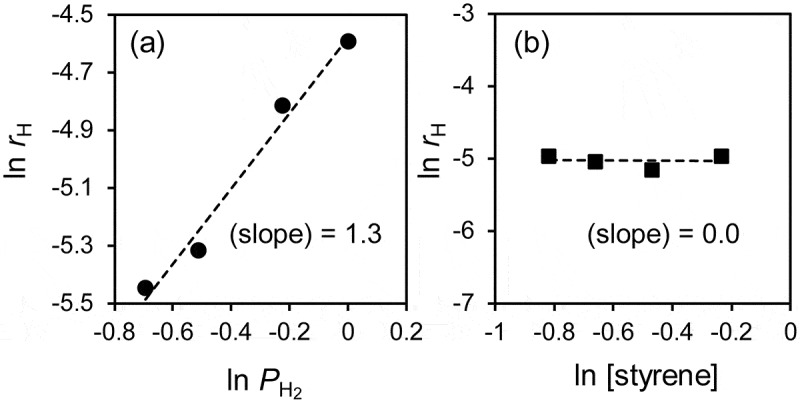
Reaction orders of H_2_ pressure and styrene concentration.

Finally, we performed a kinetic study to understand the rate-determining step of this reaction and clarify the origin of the large KIE. Figure 5 shows the dependence of the reaction rates on the (a) hydrogen pressure (*P*_H2_) and the (b) styrene concentration ([S]) in the styrene hydrogenation over RhPb_2_/SiO_2_. RhPb_2_/SiO_2_ displayed a first-order relationship with *P*_H2_ and a zero-order dependence on [S], suggesting that the styrene adsorption is saturated, and the hydrogen chemisorption is the rate-determining step of hydrogenation. The zero and first reaction orders have also been reported in the relevant catalytic systems of the liquid-phase hydrogenation of phenylacetylene or styrene [33–35], where the reactant adsorption is considered so strong. Therefore, the large KIE observed is likely originated from the dissociative adsorption of H_2_ and D_2_ onto the Rh-based intermetallic compounds. We also performed a DFT calculation for H_2_ dissociation process over the clean Rh-based surfaces. However, the activation barriers were very low (<0.08 eV, Table S1 and Figure S3). This result indicates that, under the actual reaction condition, H_2_ dissociation is hindered strongly by the adsorbed alkene molecules with high coverages. Note that there was no significant difference in the activation energy when we used the PBE functional instead of the rev-vdW-DF2 functional. Considering that the KIE value depends strongly on the phase of the intermetallic compounds and the molecular shape of adsorbates, a certain structural factor should play a key role on the large KIE. On the basis on this discussion, we propose a possible interpretation as follows. A hydrogen molecule accesses the RhPb_2_ surface covered with alkene and reaches the Rh active site, followed by dissociation. This process could be inhibited by the steric hindrance of adsorbate alkenes and/or large Pb atoms; therefore, the corresponding energy barrier and the path width differ depending on the structures of the surface and the adsorbate molecule. A large KIE might be possible when the energy barrier is significantly high for the conventional thermal process, and quantum tunneling is allowed. The Rh-based intermetallic compounds with Pb and Sb probably have appropriate surface structures that allow such a specific reaction environment and process.

## 4. Conclusions

We have prepared a series of Rh-based intermetallic compounds supported on silica and investigated their catalytic properties in the hydrogenation of unsaturated hydrocarbons. The reaction rate of hydrogenation when using H_2_ or D_2_ as a hydrogen source differed strongly, depending on the intermetallic phase. RhPb_2_/SiO_2_ exhibited an extraordinarily high KIE value of 91 in the styrene hydrogenation, which has never been reported in catalytic reactions at room temperature. This catalyst also showed high KIE values in other unsaturated hydrocarbons, such as phenylacetylene and cyclohexene. The rate-determining step of the styrene hydrogenation over RhPb_2_/SiO_2_ was found to be the dissociative adsorption of H_2_ or D_2_ on the catalyst surface covered with styrene. The large KIE observed probably originated from the quantum tunneling that occurs at the hydrogen adsorption process with the aid of the specific surface structure of the intermetallic compound and adsorbate alkene.
