# Supplementary material for: Extraordinarily large kinetic isotope effect on alkene hydrogenation over Rh-based intermetallic compounds
Source: Sci Technol Adv Mater. 2019 Jul 11;20(1):805–12. doi: 10.1080/14686996.2019.1642139 (PMC6711132; doi:10.1080/14686996.2019.1642139)
Supplement: Supplemental Material [file TSTA_A_1642139_SM0345.docx]

**Supporting Information**

Extraordinarily large kinetic isotope effect on

alkene hydrogenation over

Rh-based intermetallic compounds

Shinya Furukawa, ,*^,a,b^ Pingping Yi,^b^ Yuji Kunisada,^c^

and Ken-ichi Shimizu^a,b^

^a^ *Institute for Catalysis, Hokkaido University, N-21, W-10, Sapporo 001-0021, Japan*

^b^ *Elements Strategy Initiative for Catalysts and Batteries,*

*Kyoto University, Katsura, Kyoto 615-8520, Japan*

^c^ *Center for Advanced Research of Energy and Materials, Faculty of Engineering,*

*Hokkaido University, Sapporo 060-8628, Japan*

*E-mail: furukawa@cat.hokudai.ac.jp,*

*Tel: +81-11-706-9162, Fax: +81-11-706-9163*

Table S1. Adsorption (*E*_ad_) and dissociation (*E*_dis_) energies of H_2_ on Rh-based surfaces.

|  | *E*_ad_ (eV) | | *E*_dis_ (eV) | |
| --- | --- | --- | --- | --- |
|  | H_2_ | D_2_ | H_2_ | D_2_ |
| Rh | −0.049 | −0.049 | 0.013 | 0.011 |
| RhIn | −0.028 | −0.029 | 0.076 | 0.074 |
| RhPb_2_ | −0.046 | −0.049 | 0 | 0 |


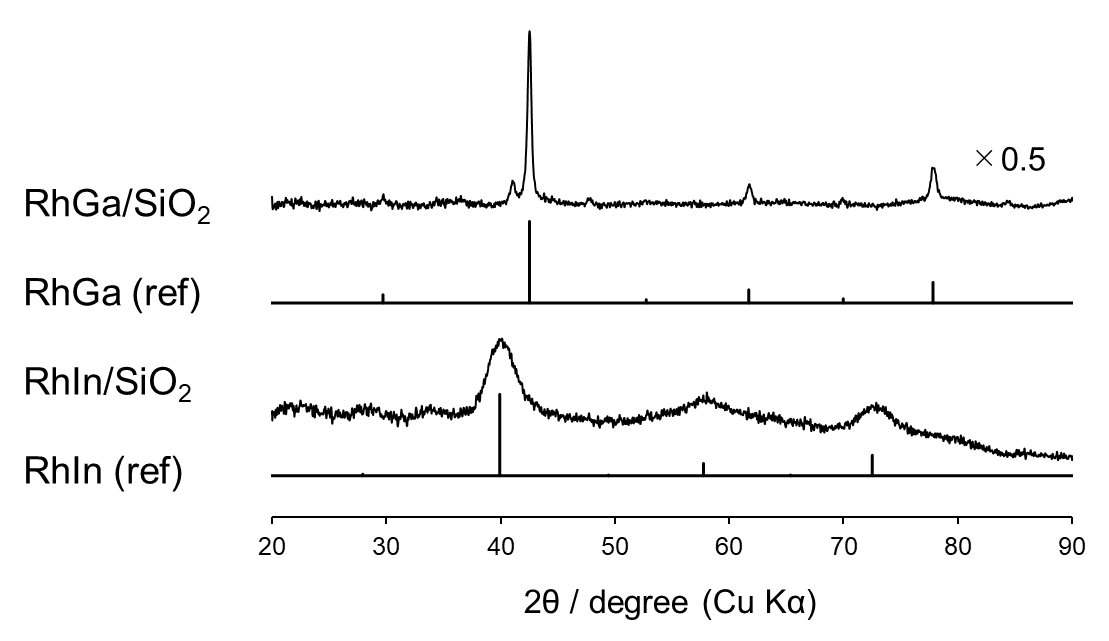


Figure S1. XRD patterns of RhGa/SiO_2_ and RhIn/SiO_2_. References are shown as black vertical lines. The desired intermetallic phases were observed with high phase purities.


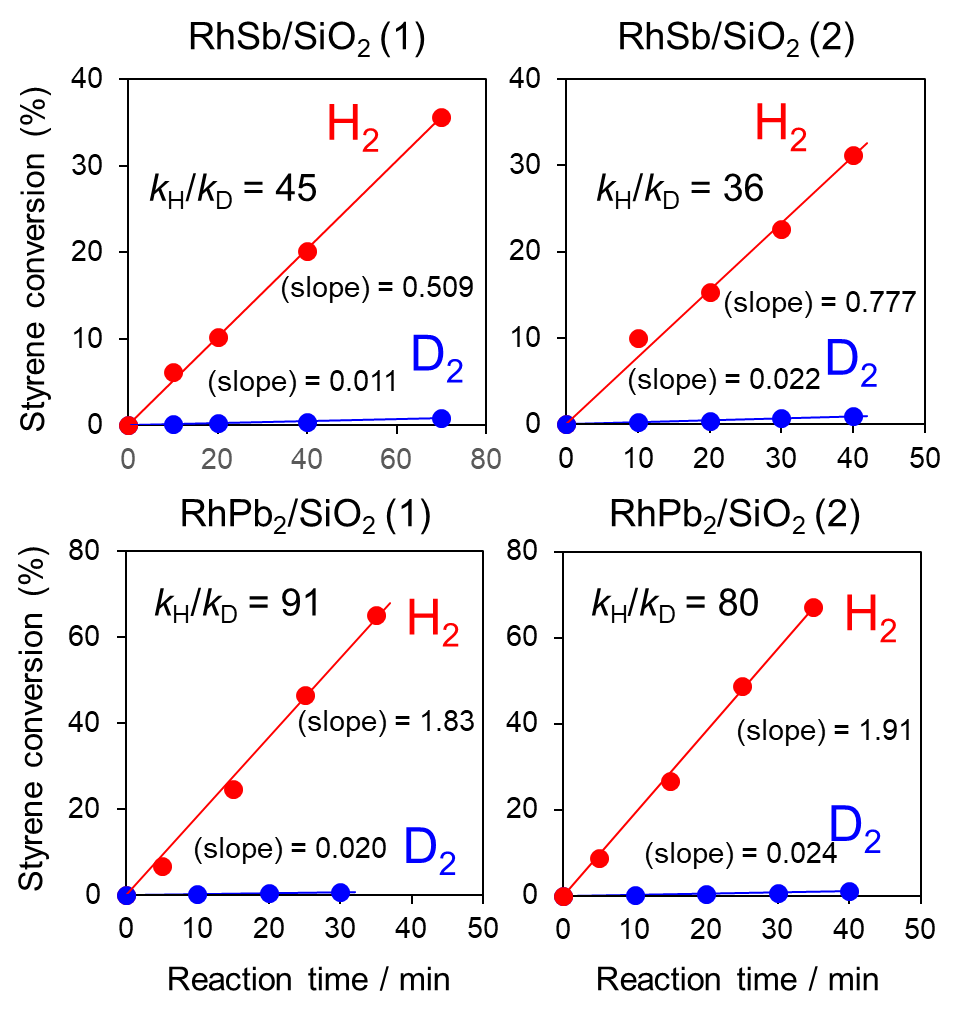


Figure S2. Time-course of styrene conversion in styrene hydrogenation over RhSb/SiO_2_ and RhPb_2_/SiO_2_ catalysts when H_2_ (red) or D_2_ (blue) was used as a hydrogen source.


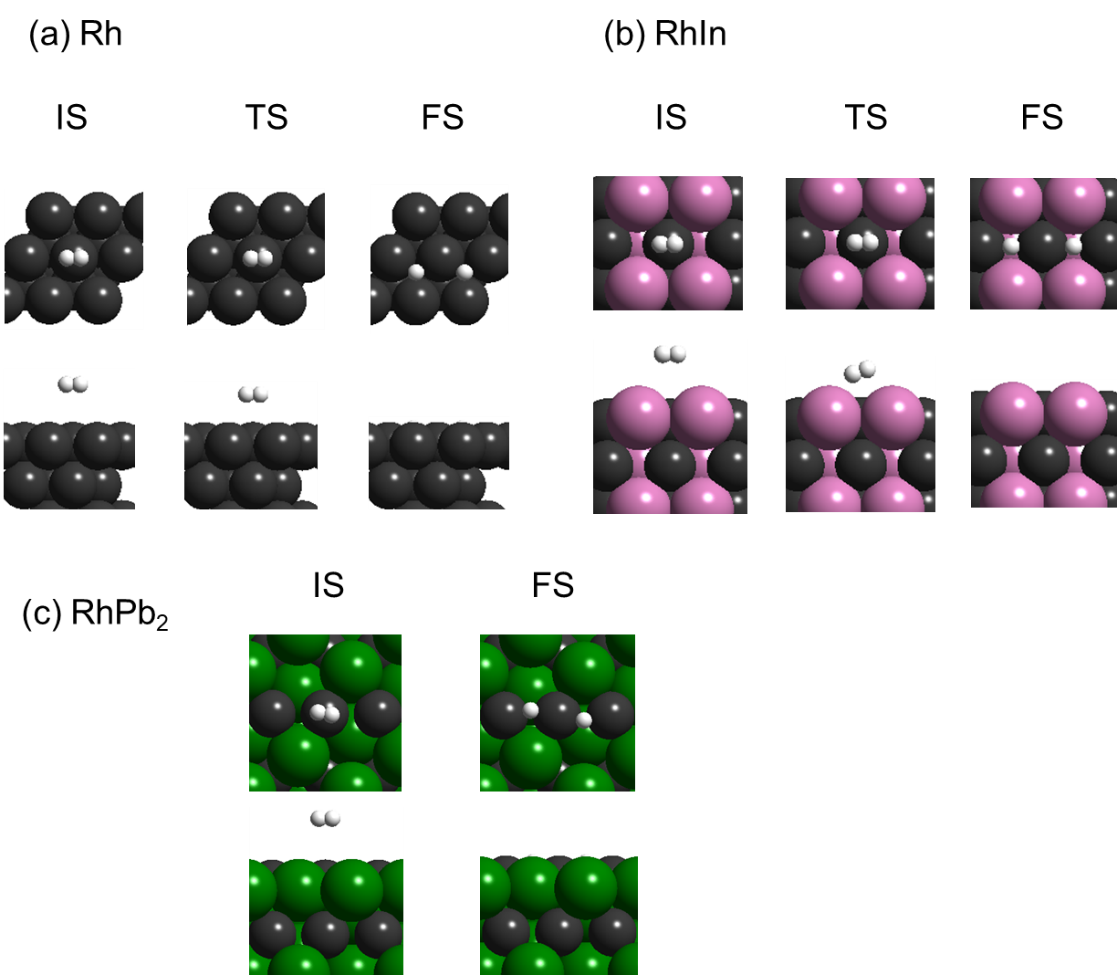


Figure S3. Structures of initial (IS: H_2_ molecule), transition (TS), and final (FS: two H atoms) states during H_2_ activation process over (a) Rh(111), (b) RhIn(110), and (c) RhPb_2_(100) surfaces.


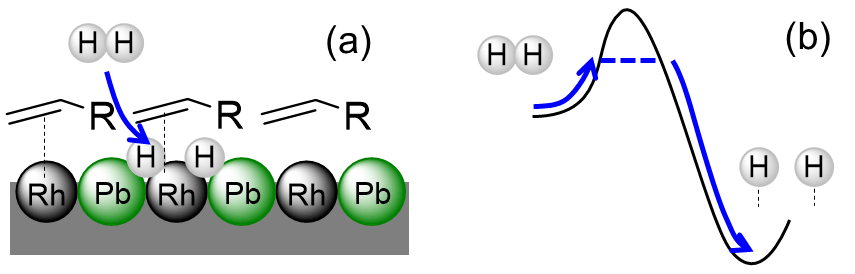


Figure S4. (a) Schematic illustration and (b) energy diagram of hydrogen dissociative adsorption on RhPb_2_ covered with alkene. The dotted line in (a) indicates adsorption of the C=C moiety on the Rh site.
